# Renal Cell Tumor and Cystic Lung Disease: A Genetic Link for Generalists to Be Aware of

**DOI:** 10.7759/cureus.43572

**Published:** 2023-08-16

**Authors:** Cedric Koh, Marc Wong, Sok Boon Tay

**Affiliations:** 1 Department of Internal Medicine, Sengkang General Hospital, Singapore, SGP; 2 Department of Respiratory Medicine, Sengkang General Hospital, Singapore, SGP

**Keywords:** renal tumors, birt-hogg-dubé, cystic lesions of the lung, carcinomas renal cell, birt-hogg-dubé syndrome

## Abstract

Birt-Hogg-Dubé syndrome (BHDS) is a rare autosomal dominant condition characterized by multiple pulmonary cysts, fibrofolliculomas, and renal cell carcinoma. The typical presentations leading to diagnosis include fibrofolliculomas and spontaneous pneumothoraxes. We present a case of a 52-year-old Chinese male who was diagnosed with BHDS after the incidental pickup of an echogenic heterogenous lesion on an abdominal ultrasound done to investigate an abnormal liver function test. The presence of renal cell carcinoma with cystic pulmonary disease should prompt the clinician to consider the diagnosis of BHDS. Knowledge of extrapulmonary findings of common cystic lung diseases may contribute to improved diagnosis of this condition.

## Introduction

First documented in the 1970s, Birt-Hogg-Dubé syndrome (BHDS) is caused by a mutation in the folliculin (FCLN) coding gene. Pulmonary cysts presenting with spontaneous pneumothoraxes and fibrofolliculomas are the most typical manifestations [1,2]. Renal cell carcinoma (RCC), while not as common, remains the most serious manifestation of the condition. In this paper, we report on an incidental renal tumor discovered during an investigation for elevated transaminases, leading to the diagnosis of BHDS. This is significant as it is a rare syndrome with potentially serious complications. We hope we can contribute to medical education and better diagnosis of this condition by highlighting the association between renal cell tumors and cystic lung disease.

## Case presentation

We report a case of a 52-year-old male Chinese patient with a background of hyperlipidemia and nonalcoholic fatty liver disease (NAFLD), with a family history of nasopharyngeal carcinoma. He had no prior exposure to industrial chemicals in his line of work. An abdominal ultrasound (US) was performed due to worsening liver function tests (alanine transaminase [ALT]: 76 U/L, aspartate transaminase [AST]: 40 U/L, alkaline phosphatase [ALP]: 104 U/L, gamma-glutamyl transferase [GGT]: 86 U/L) during routine follow-up for NAFLD in primary care. An echogenic heterogeneous focus at the interpolar region measuring 1.8 cm × 1.8 cm × 1.6 cm with minimal internal and peripheral vascularity was found.

**Figure 1:**
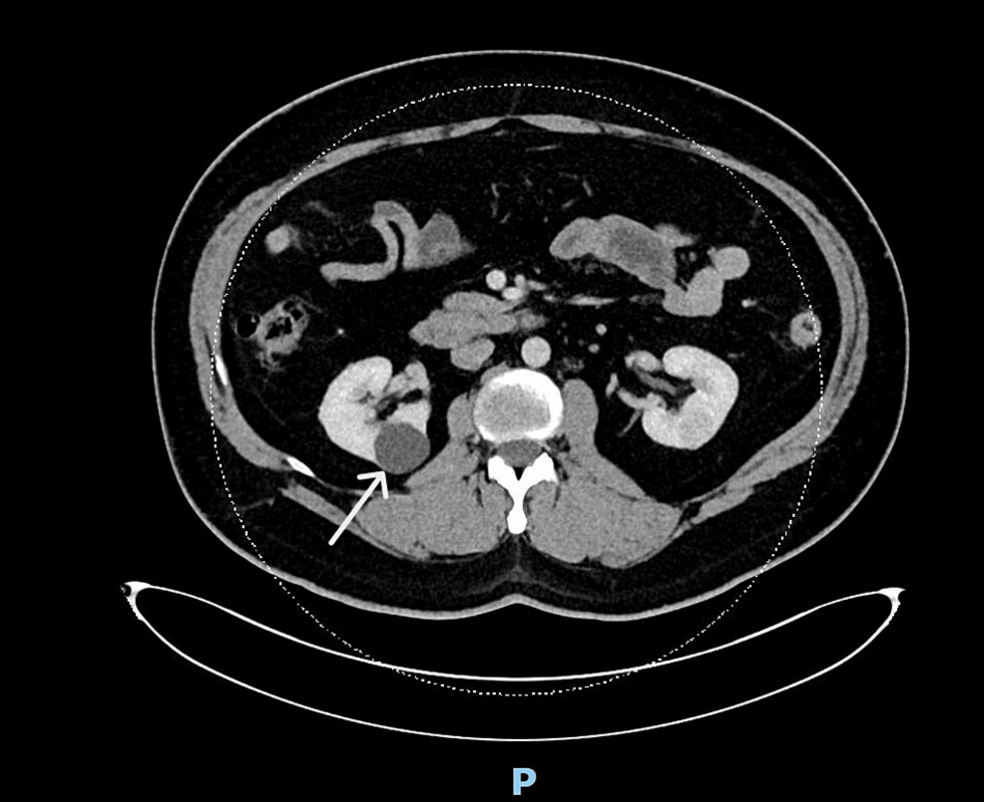
CT scan of the abdomen showing homogeneously enhancing, well-circumscribed, round lesion (arrow) in the interpolar region of the left kidney. CT, computed tomography

A computed tomography (CT) scan of his abdomen and pelvis revealed a homogenously enhancing, well-circumscribed, round lesion in the interpolar region of the left kidney measuring 2.0 cm × 1.9 cm, with an attenuation of 63 Houndsfield unit (HU) on the noncontrast study and 111 HU on the nephrogenic phase (Figure 1), suggestive of an RCC. There was no invasion into the perinephric fascia, and the left renal vein and inferior vena cava were found to be patent. Of note, there were also scattered pulmonary cysts in both lung bases and a 7 mm pulmonary nodule in the right lower lobe.

**Figure 2:**
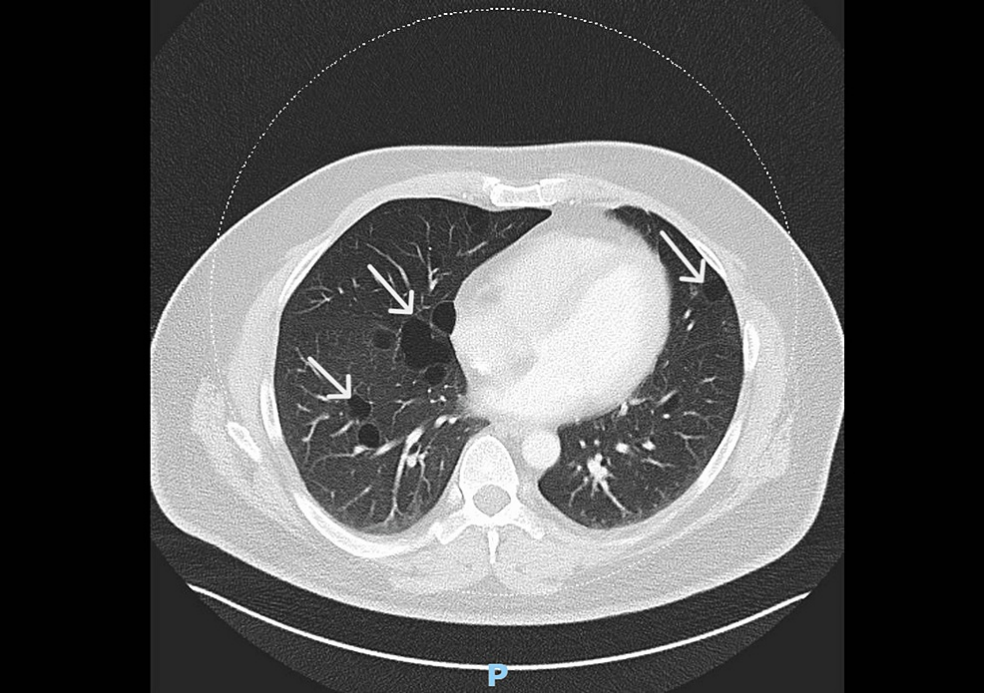
CT scan of the thorax showing multiple thin-walled cysts (arrows) in both lungs of varying sizes CT, computed tomography

He underwent a dedicated CT thorax that showed multiple thin-walled cysts in both lungs of varying sizes with no definite zonal/lobar predilection measuring up to 3.9 cm × 1.9 cm in the medial basal of the right lower lobe and 3.2 cm x 2.3 cm in the inferior lingula (Figure 2). The pulmonary nodule was noted to be stable at 7 mm. A US-guided renal biopsy was performed and histology confirmed the diagnosis of clear cell renal carcinoma. The patient subsequently successfully underwent cryoablation of his RCC.

The connection between multicystic lung disease and RCC raising the possibility of BHDS was not recognized initially. It was only during the follow-up scan for his pulmonary nodule that this possibility was raised by the reporting radiologist when supplementing the history of RCC. The presence of RCC with multiple pulmonary cysts raised the possibility of BHDS. He had skin tags over his back but no fibrofolliculomas. His CT imaging was discussed and found to be consistent with BHDS. The patient was recommended for and underwent genetic testing and was found to be positive for a c.1432+1G>C splice mutation in the FLCN gene, thus confirming the diagnosis of BHDS.

## Discussion

To date, there are a few reported cases of BHDS with RCC as the presenting problem [3]. This case describes an uncommon but serious presentation through the incidental discovery of a clear cell carcinoma in the general medicine clinic while investigating the cause of elevated transaminitis.

Cystic lung diseases are not commonly encountered in the general medicine service. The association between RCC and cystic lung disease was highlighted during interdisciplinary discussions, which led to the diagnosis of BHDS. Furthermore, this patient did not present with the more common dermatological and pulmonary manifestations. Hence, this illustrates the importance of knowing the common radiological patterns and extrapulmonary manifestations of cystic lung diseases. It is important to clinch the diagnosis of BHDS as there are implications for subsequent follow-up and genetic screening for first-degree family members.

The most common manifestations of BHDS are fibrofolliculomas or spontaneous pneumothoraxes. Reported cases of Chinese families with BHDS have shown that the most common manifestations are pulmonary cyst (92%), spontaneous pneumothorax (71%), skin lesions (18%), and renal tumors (4%)[4]. Similarly, Toro et al. [5] reported that among BHDS families in the United States, 90% of families had individuals with fibrofolliculomas, 84% of patients had lung cysts, 38% of patients had a spontaneous pneumothorax, and 34% of patients had renal tumors. Due to the propensity of cysts to rupture, affected individuals are predisposed to spontaneous pneumothorax with a high recurrence rate. There is an estimated 50-fold higher risk of pneumothorax in BHDS patients than in the general population [6]. While there seem to be differences in the prevalence of manifestations among reported Chinese and American families, dermatological and pulmonary manifestations tend to dominate.

Fewer than 5% of RCCs are thought to be from a hereditary syndrome [7]. In sporadic RCC, clear cell carcinomas are the most common subtype. This contrasts with those associated with BHDS, where mixed oncocytic/chromophobe tumors are the most common [8]. Renal chromophobe/oncocytoma is in the diagnostic criteria proposed by both Menko et al. [9] and Gupta et al. [10] for BHDS. Tumors in BHDS tend to occur at a younger age (mean age 51 years), present as multiple or bilateral tumors, and have less tendency to metastasize [11]. Surgical resection of the tumor remains the mainstay of treatment in most cases. In this case report, cryoablation was carried out as the tumor was small and detected early. This case also highlights the need for recognition of risk factors such as early onset, family history of RCC, and multifocal disease at diagnosis [7] as it may suggest a hereditary syndrome.

Pulmonary cysts described in BHDS may be oval, round, lenticular, or irregular in shape and are often smaller than 1 cm. Larger cysts are also possible and can occur concurrently with the smaller ones. They also tend to involve the basal aspects of the lung [12]. A list of key features differentiating BHDS and other commonly encountered cystic lung diseases is provided in Table 1.

**Table 1:** Key comparisons between BHDS and other commonly encountered cystic lung diseases. Radiological features are from a study by Lee et al. [12]. BHDS, Birt-Hogg-Dubé syndrome; CT, computed tomography

Disease	Radiological features on CT	Extrapulmonary features	References
Birt-Hogg-Dubé	Lower lobe predominant	Fibrofolliculomas	Jensen et al. (2017) [1]
	Round, elliptical or lenticular in shape	Renal cell carcinoma: mixed oncocytic/chromophobe tumor	Pavlovich et al. (2002) [8]
	Lenticular in shape		
	Surrounding lung often normal		
Lymphangiomyomatosis	Diffuse lung involvement	Tend to occur in women of reproductive age	Ryu et al. (2005) [13]
	Oval, round, smaller and more uniform in shape	Renal angiomyolipoma	
	Small centrilobular nodules, interlobular septal thickening, and areas of ground-glass opacities in the surrounding lung	Facial angiofibroma, subungal fibroma, and hypomelanotic macules as part of tuberous sclerosis complex	Webb et al. (1996) [14]
Pulmonary Langerhans cell histiocytosis	Upper lobe predominant	Young smoking adults	Tazi (2006) [15]
	Bizarre-shaped thin- and thick-walled cysts	Lytic bone lesions	
	Peribronchial stellate nodules in the surrounding lung	Pituitary involvement leading to diabetes insipidus	
		Eczematous rash	Newman et al. (2006) [16]
Lymphocytic interstitial pneumonitis	Lower lobe predominant	Associated with human-immunodeficiency-virus-infected patients	van Zyl-Smit et al. (2015) [17]
	Thin walled and typically small and located within areas of ground-glass opacities	Rheumatic diseases like Sjögren’s syndrome and systemic lupus erythematosus	Gupta et al. (2015) [18]

## Conclusions

The presence of RCC with cystic pulmonary disease should prompt the clinician to consider the diagnosis of BHDS. It is characterized by distinct genetic, clinical, and imaging features and may involve several organs, including the lung, skin, and kidneys. Knowledge of this syndrome is crucial for the diagnostic and therapeutic approach to cystic lung diseases and may contribute to improving the diagnosis of this rare condition.

When BHDS is suspected, a multidisciplinary approach involving respiratory physicians, radiologists, geneticists, urologists, and pathologists is needed to confirm the diagnosis and implement the appropriate follow-up. When BHDS has been identified in an index patient, a genetic workup of family members is another important step in the management of this autosomal dominant disorder.
